# CRIME-RELATED PHYSICAL INJURIES AMONG OLDER ADULTS IN THE UNITED STATES: FINDINGS FROM 2015 NIBRS DATA

**DOI:** 10.1093/geroni/igz038.1686

**Published:** 2019-11-08

**Authors:** Gerson Galdamez, Zach Gassoumis

**Affiliations:** 1 University of Southern California, Los Angeles, California, United States; 2 Leonard Davis School of Gerontology, University of Southern California, Los Angeles, California, United States

## Abstract

Approaches to addressing crimes within the justice system typically do not differentiate between younger and older adults. However, approaches to addressing elder abuse cases—which involve older adult victims exclusively—often rely on the preconception that abuse can only occur if a visible physical injury exists. This preconception is driven in part by perceptions of frailty and ease-of-injury in older, vulnerable victims. We aimed to quantify the prevalence of physical injuries among older victims of violent crimes. We used data reported by the U.S. National Incidence-Based Reporting System (NIBRS) in 2015 to quantify the frequency of reported crimes which resulted in visible, physical injuries to victims aged 60+. Logistic regression was used to determine effects of age, race/ethnicity, type of crime, relationship to offender, and victim locality on physical injury from crime. Among 1,373,417 crime victims, 80.63% of older adults (60+) sustained an injury, as opposed to 65.17% of younger adults (<60). The proportion of individuals who showed physical injuries consistently increased with age until age 90. Knowing the offender was associated with higher odds of sustaining a visible injury (OR 1.47, 95% CI 1.38-1.58). Although older adults show higher risk of visible physical injuries from violent crimes than younger adults, it is possible for a crime against an older adult to have occurred without a visible injury. Medical and criminal justice practitioners should utilize this evidence on elder crime victimization to aid in expert witness testimony and other activities related to justice in crimes against older adults.

